# Effectiveness of the Advanced Practice Nursing interventions in the patient with heart failure: A systematic review

**DOI:** 10.1002/nop2.847

**Published:** 2021-03-10

**Authors:** Javier Ordóñez‐Piedra, Jose Antonio Ponce‐Blandón, Jose Miguel Robles‐Romero, Juan Gómez‐Salgado, Nerea Jiménez‐Picón, Macarena Romero‐Martín

**Affiliations:** ^1^ Facultad de Enfermería Fisioterapia y Podología Universidad de Sevilla Sevilla Spain; ^2^ Centro Universitario de Enfermería de Cruz Roja Universidad de Sevilla Sevilla Spain; ^3^ Departamento de Sociología Trabajo Social y Salud Pública Universidad de Huelva Huelva Spain; ^4^ Universidad Espíritu Santo Guayaquil Ecuador; ^5^ Present address: Departamento de Enfermería Universidad de Huelva Huelva Spain

**Keywords:** advanced nurse practitioners, advanced practice, cost of care, death and dying, heart disease, nurse, nursing practice, quality of life, systematic review, unplanned readmission

## Abstract

**Rationale and Aim:**

Advanced Practice Nurse (APN) is a specialist who has acquired clinical skills to make complex decisions for a better professional practice. In the United States, this figure has been developed in different ways, but in some European countries, it is not yet fully developed, although it may imply a significant advance in terms of continuity and quality of care in patients with chronic or multiple pathologies, including cardiac ones and, more specifically, heart failure (HF). The follow‐up of HF patients in many countries has focused on the medical management of the process, neglecting all the other comprehensive health aspects that contribute to decompensation of HF, worsening quality indicators or patient satisfaction, and there are not updated reviews to clarify the relevance of APN in HF, comparing the results of APN interventions with doctors clinical practice, since the complexity of care that HF patients need makes it difficult to control the disease through regular treatment. For this reason, this systematic review was proposed in order to update the available knowledge on the effectiveness of APN interventions in HF patients, analysing four PICO questions (Patients, Interventions, Comparison and Outcomes): whether APN implies a reduction in the number of hospital readmissions, if it reduces mortality, if it has a positive cost‐benefit relationship and if it implies any improvement in the quality of life of HF patients.

**Design and Methods:**

A systematic review was performed based on the PRISMA statement, searching at four databases: PubMed, CINAHL, Scopus and Cuiden. Articles were selected based on the following criteria: English/Spanish language, up to 6 years since publication, and original quantitative studies of experimental, quasi‐experimental or observational character. Papers were excluded if they do not comply with CONSORT or STROBE checklists, and if they had not been published in journals indexed in JCR and/or SJR. For the analysis, two separate researchers used the Cochrane Handbook form for systematic reviews of intervention, collecting authorship variables, study methods, risks of bias, intervention and comparison groups, results obtained, PICO question or questions answered, and the main conclusions.

**Results:**

A total of 43,754 patients participated in the 11 included studies for the development of this review, mostly from United States and non‐European countries, with a clearly visible lack of European publications. Regarding the results related to first PICO question, researches reviewed proved that APN implied a reduction in the number of hospital readmissions in patients with heart failure (up to 33%). Regarding the second question, mortality was always lower in groups assisted by APN versus in control groups (up to 7.8% vs. 17.7%). Regarding the third question, APN was cost‐effective in this type of patient as the cost reduction was eventually calculated in 1.9 million euros. Regarding the last question, quality of life of patients who have been cared for by an APN had notoriously improved, although one of the papers concluded that no significant differences were found. All the questions addressed obtained a positive answer; therefore, APN is a practice that reduced hospital readmissions and mortality in HF patients. The cost‐effectiveness is much better with APN than with usual care, and although the quality of life of HF patients seems to improve with APN, more studies are needed to support this focused on this.

**Summary box**

- Advanced practised nursing addressed at patients with heart failure has proven reduce hospital readmissions and also mortality.
- Advanced practised nursing also has positive effects on cost‐benefit and on the improvement of quality of life of heart failure patients.
- The advanced clinical competences are needed for the enhanced professional practice to improve heart failure patients’ care.

**What does this paper contribute to the wider global clinical community?**

- This review is the first comprehensive analysis of clinical trials, quasi‐experimental and observational studies with the greatest rigour and quality bringing together the best evidence that confirms the ability of advanced practical nursing to reduce hospital readmissions and mortality of HF patients.
- The verification made by this review about the impact of advanced practice nursing to improve cost‐effectiveness of care for patients with HF and their quality of life makes the development of this care system as a highly recommended task to improve the efficiency of and effectiveness of health services.
- Confirming the positive impact that advanced practice nursing can have on the results of care of patients with HF allows us to extend this model of advanced care to other chronic, multiple and/or complex pathologies.

## 1 INTRODUCTION

Advanced Practice Nursing was defined in 2002 by the International Council of Nurses as “a specialist nurse who has acquired the expert knowledge base, the abilities to adopt complex decisions and the clinical competences needed for an enhanced professional practice, whose characteristics are given by the context or country in which the nurse is accredited to work in” (International Council of Nurses, 2008).

This same concept having been generally described, it is necessary to clarify that the concept of Advanced Practice Nursing (APN) includes different specific and well‐differentiated Nursing roles such as: Nurse Practitioners (NPs), Certified Nurse‐Midwives (CNMs), Certified Registered Nurse Anaesthetists (CRNAs) and Clinical Nurse Specialists (CNSs), always in the scope of the United States (Jansen & Zwygart‐Stauffacher, 2010). Each of these same Advanced Practice Nursing roles has its own history and defining characteristics, satisfying certain social needs inside the U.S. sociocultural setting.

In some countries of the European context, the concept of APN is not yet fully developed: only certain degree courses train for certain very concrete procedures, but never qualifying as APN to the extent we are describing in the American setting (Agencia de la Calidad Sanitaria de Andalucía. Consejería de Salud y Familias, 2019). It would be very interesting to implement this professional category in the primary care setting, an area where the need for family doctors is urgent and whose tasks can be carried out by these figures in order to take care of Heart Failure (HF) patients or other chronical patients. In order to do so, health managers, politicians and the collective of the different professions together with their leaderships should know that, to provide an adequate health care to the population as a whole, it is necessary to adopt the idea that that is precisely what matters, independently of who provides these services (Galao‐Malo, 2009).

Advanced Practice Nursing can imply a significant move forward with regards to care continuity and quality of care in a variety of patients but, especially, in patients with chronic or multiple pathologies, cardiac ones included and, more specifically, heart failure.

Heart failure has become one of the most prevalent syndromes in today´s society, being the most important cause of morbimortality and hospitalizations in developed countries. The prolongation of life in patients with pathologies traditionally considered untreatable, like myocardial infarction or valve‐related pathologies, together with a greater understanding of the cardiac physiopathology and its pharmacological and surgical treatments over the last decades, has brought about a substantial improvement in the prolongation and quality of life of the patient and, therefore, an increase in the prevalence of heart failure, as mentioned above (Eisen, 2017).

It is considered a complex syndrome that provokes cardiac output due to the reduction in the ventricular blood filling or ejection capacity due to the structure of functional cardiac disorders, resulting in a situation in which the heart is unable to sustain the tissular metabolic outputs or in which, to sustain them, compensatory filling pressures are required above the normal values (Rahko, 2013).

Complexity of care needed by these types of patients is underlined because the risk of decompensation, especially in advanced stages of the disease, making the control the disease very difficult to manage through regular treatment, which leads to HF becoming refractory, which may or may not be reversible, depending on various standardized levels of complication established by New York Heart Association (NYHA; Rostagno et al., 2000) or American College of Cardiology in combination with American Heart Association (Marín‐García, 2010).

Complexity of their care represents the fact that not all patients are able to have some self‐control over their own care, since both the variety and complexity of most treatments provided for this disease, and the particular variety of every patient (physical, psychological and psychiatric reasons) sometimes causes great confusion, leading to forgetfulness and mistakes in taking medications, for example (Bartunek & Vanderheyden, 2013).

For this, APN, in a qualified way, should train patients to detect the main signs and symptoms of the disease, and the resources they can use in the different situations that may arise as a direct consequence of the disease, like exacerbations or gradual worsening of the general patient condition, or indirect consequences such as the functioning of household or the tiredness of the role of the caregivers (Henein, 2010). Regarding the care of patients with HF, another essential nursing intervention is related to provide all the necessary information about social and personal consequences of the disease, either in terms of the appropriate lifestyles that must be carried out or in terms of aspects as varied as sexual activity, self help groups, immunization, or driving and travel advices (López‐Castro, 2019).

The lack of a nursing care model has meant that monitoring of HF patients in many countries has been focused on the medical management of the process, neglecting all the other comprehensive health aspects that contribute to the decompensation of HF, worsening quality indicators or patient satisfaction indicators. There is evidence that, working under the interdisciplinary model, advanced practice nurses reduce hospitalizations for patients with coronary heart disease. Furthermore, specialized nurses together with doctors can achieve improvements in patients with other chronical illness such as diabetes (Health Quality Ontario, 2013).

There is some evidence that CNSs and their transitional care model improve many aspects such as patient health outcomes, re‐hospitalization rates and costs, and delaying re‐hospitalization and reducing hospital stay. In addition, the work of the CNSs produces benefits not only for patients, but also for caregivers, and may even improve depression in both. Finally, they also produce benefits in women with high‐risk pregnancies and low birth‐weight children (Donald et al., 2014).

The postdischarge HF patient management by the APNs, instead of usual RNs, through telecare programmes, produces positive effects in several areas. One of them is the improvement of the quality of life of the patient, due in part to the decrease in the re‐hospitalizations so frequent of this syndrome, causing in addition, an important economic benefit for the health system, since it occurs savings both in occupancy of beds and medicines. It must be understood, therefore, that, although the salary of NPC is higher than the RN salary, in the long term the return on investment will make sense, since substantial costs would be saved in terms of re‐hospitalizations and direct health outcomes, as previously discussed (Delgado‐Passler & McCaffrey, 2006). This is palpable in another review, which specifies that patient discharge management programmes led by Nursing again produce positive results, demonstrating the potential of nursing leadership in this regard, although it is necessary a greater number of studies to relate this fact with the improvement of HF patients (Lambrinou et al., 2012).

HF patient management in an outpatient setting by APRNs is another interesting measure to analyse, since there are also studies concluding that it produces positive effects not only for patients, but also for nursing professionals themselves. However, there are not enough reviews comparing the results of APRNs interventions with doctors clinical practice, and, in addition, more review papers analysing the economic results regarding the actions of advanced practice nurses are needed (Case et al., 2010).

Thus, the experiences of recent systematic reviews have been very useful in order to contribute to a better understanding of the nursing care, in other contexts, such as the analysis of the nature of care (Romero‐Martín et al., 2019), or the analysis of the benefits of nursing prescribing (Ponce‐Blandón et al., 2020) and even to compare general advanced practice with clinical nursing (Cooper et al., 2019), to analyse the leadership competencies and attributes in advanced nursing practice (Heinen et al., 2019) or about educational preparedness of advanced clinical practitioners (Dover et al., 2019). However, specifically to clarify the relevance of APN in HF, we must refer to the review carried out by Case et al. (2010), the only more recent experience in this line of care directed towards HF.

This complex syndrome being included in the chronic pathologies as already mentioned, Advanced Practice Nursing could imply one of the care solutions in certain sociodemographic environments for these patients. Then, due to the described lack of knowledge and in order to verify if these positive results of applying APN Nursing to the care process of patients with HF are indeed a reality and if they are applicable to the context of a Health National System's health model in which APN is not a reality, it is necessary to search for the latest evidence available in the literature, so as to offer an alternative viewpoint on the real capacities and the functions that APN Nursing could undertake, where the competences provided during our University studies would be considerably more profoundly leveraged.

## 2 OBJECTIVES

The aim is to perform an updated literature review so as to know the effectiveness of the Advanced Practice Nursing interventions in the patient with heart failure, analysing if these interventions imply a result in any reduction in the number of hospital readmissions, if they produce any benefit in relation to these patients’ mortality, if they have any positive cost‐benefit ratio, and if applying the Advanced Practice Nursing procedures imply any improvement in the quality of life of the users of the health system suffering from this pathology.

In order to develop these review objectives, and applying the PICO (Patients, Interventions, Comparison, Outcomes) model for the formulation of clinical questions in the practice based on evidence (Martínez Díaz et al., 2016), the four PICO questions shown in Table 1 were established.

**TABLE 1:** Description of the PICO questions for the review

PICO question	Participants	Intervention/Comparison	Result
PICO No. 1: Do the Advanced Practice Nursing interventions imply any reduction in the number of hospital readmissions in patients with heart failure?	Nursing professionals and users of the health system.	Advanced Practice Nursing versus other health‐related professions.	Reduction or increase in the number of hospital readmissions.
PICO No. 2: Is mortality reduced by means of Advanced Practice Nursing in the patients with heart failure?	Nursing professionals and users of the health system.	Advanced Practice Nursing versus other health‐related professions.	Reduction or increase in the number of hospital readmissions.
PICO No. 3: Is Advanced Practice Nursing cost‐effective in the patient with heart failure?	Nursing professionals and users of the health system.	Advanced Practice Nursing versus other health‐related professions.	Cost‐effective or not cost‐effective.
PICO No. 4: Do these same interventions produce any improvement in the quality of life of the patient with heart failure?	Nursing professionals and users of the health system.	Advanced Practice Nursing versus other health‐related professions.	Reduction or improvement in the patient's quality of life.

## 3 METHODS

A systematic review procedure was developed during the period from March to July 2018. The PRISMA statement (Urrútia & Bonfill, 2010) has served as the structural base for this study, in such a way that the development was planned of the 27 items which compose the checklist of such statement. To that end, a review protocol was elaborated which included a clear definition of the eligibility criteria (inclusion and exclusion criteria), of the information sources to be employed, of the search strategies applied, and of the procedure for selecting the studies to be included in the review, in such a way that this protocol was applied by two reviewers with clinical, teaching and research experience and over 3 years of experience in narrative, integrative and systematic reviews. At the end of the selection process, and once consensus was reached on those matching articles in the lists of selected articles, the two reviewers collaboratively undertook the following tasks: data extraction from these articles, elaboration of the list of this data, identification of the bias risk, identification of summary measures, synthesis of the results and additional analyses.

In the selection process, carried out by the reviewers, the following inclusion and exclusion criteria were defined:

- Inclusion criteria:
  - Language: Spanish and English.
  - Year of publication of the selected studies: the search was limited to articles published after 2012.
  - Design of the selected studies: quantitative studies of experimental, quasi‐experimental or observational character.
  - Articles which answered at least one PICO question.
- Exclusion criteria:
  - Articles that are not fully available in the virtual library of the University of Seville.
  - Duplicate studies.
  - Review articles and/or not original articles.
  - Articles which did not meet the quality criteria: complying at least with 80% of the items in the CONSORT (Cobos‐Carbó & Augustovski, 2011) or STROBE (Von Elm et al., 2008) checklists, and publications in journals not indexed in JCR and/or SJR.

The databases in which the search was performed were the following: PubMed (US National Library of Medicine, National Institutes of Health), CINAHL (Cumulative Index to Nursing and Allied Health Literature), Scopus (Elsevier's Bibliographical Database) and Cuiden (Database of the Index Foundation on Health Care), since they are the most relevant information sources with the most likelihood of finding the best quality scientific papers on the theme and the objectives of the review. Although only 4 databases were consulted, in accordance with recommendations for conducting systematic reviews in the clinical setting (Linares‐Espinós et al., 2018) and in the specific field of nursing (Aromataris & Pearson, 2014), it was decided to select the most relevant databases according to the bibliography described, because, although the search in these four databases tend not to identify the same number of references, it is known that they do find a similar number of “ relevant ” studies. In any case, the procedures established for systematic reviews in The PRISMA statement (Urrútia & Bonfill, 2010) were strictly followed.

The subsequent process for the selection of the articles was exactly the same for all the databases used, using the following descriptors: “Nurse Practitioner,” “APN,” “Advanced Practice Nursing,” “Nurse‐led” and “Heart failure,” all included in MeSH (Medical Subject Headings) and in DeCS (*Descriptores en Ciencias de la Salud*, created by BIREME, Latin American and Caribbean Centre of Information in Health Sciences). Later, to complement the search, the descriptor "Clinical Nurse Specialist" was added. In the case of the Cuiden database, as it is in Spanish, the following descriptors were used: “*Enfermería de práctica avanzada*” and “*Insuficiencia cardiaca,*” both included in DeCS. The search strategy applied systematically combining the Boolean operators with the aforementioned descriptors was (“Nurse Practitioner” OR “APN” OR “Advanced Practice Nursing” OR “Nurse‐led”) AND “Heart failure” NOT “Review,” with the exception of Cuiden, in which the following strategy was applied: “*Enfermería de práctica avanzada*” AND “*Insuficiencia cardiaca*.” The search was repeated incorporating the aforementioned descriptor "Clinical Nurse Specialist" with the boolean "OR" together with the rest of the terms mentioned linked to the descriptor "APN" into the search strategy.

The first discard phase of articles was performed by reading the titles and abstracts from the articles obtained using the search strategies, following the established inclusion and exclusion criteria, excluding duplicates, articles which clearly did not use observational and/or experimental quantitative methodologies according to the description of the methodology in the abstract, and papers in which the abstract did not clearly express any answer to at least one of the PICO questions posed.

For data extraction from the selected articles in this first screening, the full texts were obtained and the form of the Cochrane Handbook for intervention systematic reviews (Cumpston et al., 2019) was undertaken in duplication and separately, collecting the variables of authorship, study methods, bias risks, intervention and comparison groups (if pertinent), results obtained (main measures), PICO question or questions answered and the main conclusions. The data extracted simultaneously by the two researchers were later compared and completed between each other, in such a way that, after this joint analysis, articles were discarded in which it was mutually agreed and confirmed that at least one inclusion or exclusion criterion was not met.

The information obtained was jointly analysed and a narrative synthesis was performed which included the result of the identification of the article (lead author/year/country), the methodological design employed, the sample of participants, the objectives of the study and the main results and its conclusions, classifying the research based on the PICO question(s) it definitely answered and excluding from the review those articles which, after analysing their full text, were found to not answer correctly at least one of the PICO questions.

Finally, a search was performed to check if the journals in which the chosen articles were published were indexed in the JCR (Journal Citation Report) and/or in the SJR (Scimago Journal & Country Rank), excluding those articles whose journal did not appear in at least one of the lists, verifying the impact factor of each of them and the corresponding quartile occupied by the journal in the year of their publication.

The review was approval by Research Ethic Committee of Red Cross Nursing School, University of Sevilla.

## 4 RESULTS

**FIGURE 1:**
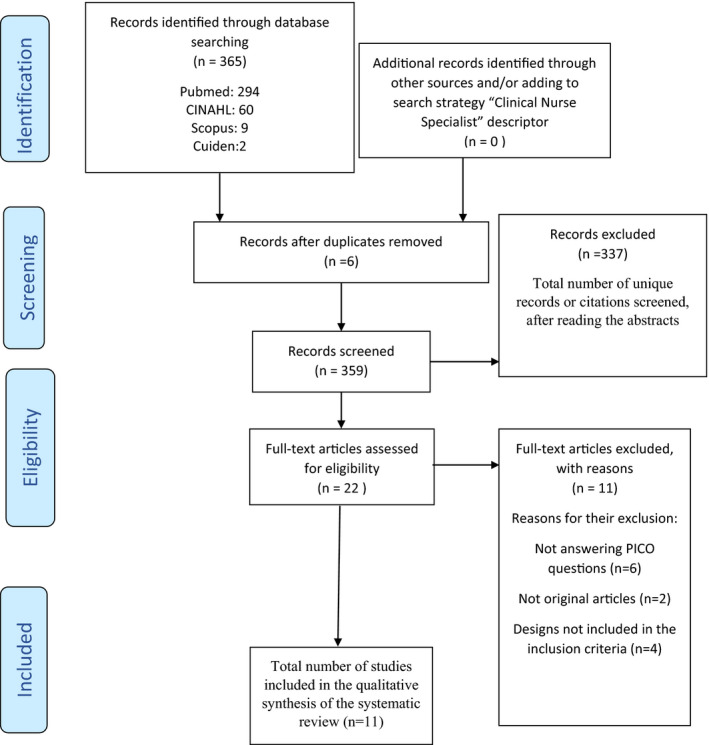
Flow diagram of the systematic review

In the preliminary search performed by the reviewers in the mentioned databases, 365 articles were obtained, 6 records being deleted due to duplicity. Subsequently, 336 records were deleted by applying the inclusion and/or exclusion criteria after analysing the abstracts, and another 11 records were also deleted after applying these same criteria but in the analysis of the full texts, thus obtaining a total of 11 articles for the qualitative synthesis of the systematic review. The flow diagram of the information through the different phases of the review, based on the PRISMA statement (Urrútia & Bonfill, 2010), can be seen in Figure 1.

All the selected articles are written in English, and more than half (*n* = 6) share their geo‐location between the U.S. and Australia, while the rest are distributed among countries as diverse as Sweden, China or Brazil. With regard to the research designs employed, most are experimental papers (*n* = 8), while two are observational (*n* = 2), with only one paper left of a quasi‐experimental character (*n* = 1). The sampling size varied considerably in the different articles included in the review, ranging from 78 patients to 38,618 patients. The articles were published between 2012 and 2017.

Regarding the impact of the selected articles, in the assessment of the impact factor and the quartiles the publications occupied in the JCR and in the SJR, the results included in Table 2 were obtained. In that table, it can be seen that there are four journals in Q1 in the JCR and 5 in Q1 in the SJR, Q3 being the lowest quartile of the journals analysed in both lists.

**TABLE 2:** Impact factor and quartile in the JCR and in the SJR of the journals in which the selected articles were published

Author(s) (year)	Journal	Impact factor		Quartile (Q)	
		JCR	SJR	JCR	SJR
Cheng et al. (2016)	Journal of Geriatric Cardiology	1.806	0.757	Q3	Q2
Stewart et al. (2016)	Circulation	19.309	8.534	Q1	Q1
De Souza et al. (2014)	European Journal of Heart Failure	6.526	3.997	Q1	Q1
Lowery et al. (2012)	Congestive Heart Failure	Does not appear	0.806	Does not appear	Q1
Ågren et al., (2013)	Journal of Clinical Nursing	1.233	0.8	Q2	Q1
Cajanding (2016)	Applied Nursing Research	1.379	0.483	Q2	Q2
Smith et al. (2015)	Journal of Cardiovascular Nursing	2.172	0.743	Q3	Q3
Kutzleb et al. (2015)	Nursing Economics	0.934	0.526	Q3	Q2
Maru et al. (2018)	European Journal of Cardiovascular Nursing	2.763	0.929	Q1	Q1
Raji et al. (2016)	Journal of Primary Care & Community Health	Does not appear	0.598	Does not appear	Q2
Agrinier et al. (2013)	International Journal of Cardiology	6.175	0.944	Q1	Q2

Referring to the answers to the PICO questions, a total of 9 articles addressed the influence of Advanced Practice Nursing in the number of hospital readmissions in these patients (PICO No. 1; Agrinier et al., 2013; Cheng et al., 2016; Kutzleb et al., 2015; Lowery et al., 2012; Maru et al., 2018; Raji et al., 2016; Smith et al., 2015; De Souza et al., 2014; Stewart et al., 2016), five of the selected papers addressed the influence of these interventions in the potential reduction of the patients’ mortality (PICO No. 2; Agrinier et al., 2013; Cheng et al., 2016; Lowery et al., 2012; De Souza et al., 2014; Stewart et al., 2016), up to seven papers evaluated the cost‐effectiveness ratio of the advanced practice in patients with heart failure (PICO No. 3; Ågren et al., 2013; Agrinier et al., 2013; Kutzleb et al., 2015; Lowery et al., 2012; Maru et al., 2018; Raji et al., 2016; Smith et al., 2015) and a total of three studies evaluated the influence of Advanced Practice Nursing in the quality of life of these patients (PICO No. 4)(Ågren et al., 2013; Cajanding, 2016; Smith et al., 2015).

In relation to the number of hospital readmissions, all the articles which addressed PICO question No. 1 conclude that the Advanced Practice Nursing interventions have an impact on the reduction of such a number in patients with heart failure (Agrinier et al., 2013; Cheng et al., 2016; De Souza et al., 2014; Kutzleb et al., 2015; Lowery et al., 2012; Maru et al., 2018; Raji et al., 2016; Smith et al., 2015; Stewart et al., 2016). The readmission ratio in some studies is much lower in the patients with heart failure assisted by Advanced Practice Nursing interventions, as shown by the Smith et al. study (2015), which verified a 33% reduction in the readmission ratio. Certain studies, like Cheng et al. (2016), place the risk of hospital readmission in patients not assisted by Advanced Practice Nursing more than seven times above that of those assisted by this care profile (OR = 7.40). In other studies, the differences are not so pronounced, although they remain significant, as in the case of the Stewart et al. study (2016), with a rate of 0.22 readmissions versus 0.36 in those not assisted by the advanced practice, of the De Souza et al. study (2014), with 32.4% of readmissions versus 38.8% in patients without the advanced practice interventions, or of the Lowery et al. study (2012), which verified a reduction of 0.12% in the number of readmissions in the first year versus 0.22% in the patients assisted by Advanced Practice Nursing.

Regarding the PICO question No. 2, about the impact of Advanced Practice Nursing in the reduction of the mortality of patients with heart failure, four of the articles of which address this issue (Cheng et al., 2016; De Souza et al., 2014; Lowery et al., 2012; Stewart et al., 2016) agree that the patients’ mortality decreases, in a moderate or moderately high rate, but always in a more decreased direction, but always obtaining positive results in this aspect. Only one of the articles addressing this issue (Agrinier et al., 2013), concluded that mortality in the population who has access to the programme that the article deals with is equal to that of the population in which this programme has not been incorporated. The mortality figures considered in the papers are over 8.5% in patients assisted by the advanced practice programme versus 14% (De Souza et al., 2014), 7.8% versus 17.7% in the first year and 14.5% versus 27.6% in the second year (Lowery et al., 2012), or a mortality rate of 0.50 versus 0.88 (Stewart et al., 2016).

With regards to the cost‐effectiveness ratio of Advanced Practice Nursing in the patient with heart failure, five of the seven articles which answered this PICO question conclude that Advanced Practice Nursing in the patient with heart failure is cost‐effective (Agrinier et al., 2013; Kutzleb et al., 2015; Lowery et al., 2012; Raji et al., 2016; Smith et al., 2015), always with differences in the level of benefit it produces, although in a study conducted with 312 patients (Kutzleb et al., 2015), the cost of Advanced Practice Nursing is calculated a little above USD 300,000 versus more than USD 1,000,000 in the control group or, in the Agrinier et al. paper (2013), it is calculated that Advanced Practice Nursing applied in 1,222 patients implied a cost reduction of more than 1.9 million euros. Faced with this data, other studies conclude that the interventions they analysed in their respective papers (Ågren et al., 2013; Maru et al., 2018) are not cost‐effective in the time frame they have been developed, with the need for a longer establishment period to allow for a thoughtful and correct assessment.

Finally, with respect to the PICO question referring to the impact of Advanced Practice Nursing on the quality of life of the patients with heart failure, of which three papers were identified, whereas two of the authors (Cajanding, 2016; Smith et al., 2015) point out that the quality of life of the patients who have been in the intervention programmes has notoriously improved, one of the three papers (Ågren et al., 2013) has concluded that no significant differences were found in the quality of life of the patients subjected to the intervention programme compared to that of those who did participate in them.

The details of all this information, together with the main results of the descriptive analysis performed on the articles included in the review, are summarized in Table 3.

**TABLE 3:** Descriptive analysis summary of the articles included in the review

Lead author/Year/ Country	Methodology	Sample	PICO	Objective	Main results	Conclusions	CONSORT/STROBE
Cheng HY/2016/China	Analytical, retrospective, observational	78 patients	1,2	To evaluate the effects produced by a clinic run by nurses on hospital readmission and mortality in patients with heart failure.	The patients who did not go to the clinic had a higher risk of hospital readmissions [OR: 7.40; *p* < .01] and higher mortality (*n* = 14) than those who did go to the clinic (*n* = 4).	The fact that the nurses run the clinic reduces the number of hospital readmissions and the patients’ mortality.	19/22
Stewart S/2016/Australia	Experimental: a randomized clinical trial	1,226 patients	1,2	To determine the effects of a home intervention conducted by nurses and adapted to patients with chronic cardiac pathologies.	The patients who received home care presented lower mortality (0.50 versus 0.88) and fewer hospital readmissions (0.22 versus 0.36).	The home interventions conducted by nurses reduce both mortality and the number of hospital readmissions.	21/25
De Souza EN/2014/Brazil	Experimental: a randomized clinical trial	252 patients	1,2	To check if an intervention strategy conducted by nurses can be beneficial for the patients with heart failure.	The patients in the intervention group presented fewer hospital readmissions (32.4% versus 38.8%) and lower mortality (8.5% versus 14.5%).	The home interventions conducted by nurses reduce the patient's mortality and the number of hospital readmissions.	19/25
Lowery J/2012/U.S.	Quasi‐experimental	969 patients	1,2,3	To compare the results obtained by means of an NP leadership model in chronic diseases to those obtained with a medical leadership model.	In the first year, hospital readmission in the intervention group decreased (0.12% versus 0.22%), with the same figures in the second year. Mortality also decreased, both in the first year (7.8% versus 17.7%) and in the second year (14.5% versus 27.6%) in the intervention group. The NP model is more cost‐effective.	The NP leadership model reduces the number of hospital readmissions and the patient's mortality. This model is more cost‐effective than the traditional one conducted by physicians.	23/25
Ågren S/2013/Sweden	Experimental: a randomized clinical trial	155 patients	3,4	To determine the cost‐benefit ratio of an education programme led by nurses in patients with heart failure.	No significant differences were found between the intervention and control groups as regards quality of life. It did not turn out to be sufficiently cost‐effective; however, it could be if the study was extended to more than 1 year.	The education programme led by nurses did not produce the desired results as regards the quality of life of the patient. It did not turn out to be cost‐effective in the period in which it was implemented, more time being needed.	24/25
Cajanding RJ/2016/ Philippines	Experimental: a randomized clinical trial	100 patients	4	To know the effectiveness of a cognitive‐behavioural programme led by nurses in patients with heart failure.	The quality of life of these patients was increased and that was verified with three different tests. In all of them, higher quality of life values were obtained in the intervention group.	The cognitive‐behavioural programme led by nurses produced an increase in the quality of life of the patient.	22/25
Smith CE/2015/U.S.	Experimental: a randomized clinical trial	198 patients	1,3,4	To evaluate the effects of a nursing leadership intervention in patients with heart failure.	The readmission rate was reduced by 33%, improving the quality of life of the patient and obtaining a cost‐effective result.	The nursing leadership intervention reduced the number of readmissions, besides improving the quality of life of the patient and proving to be cost‐effective.	24/25
Kutzleb J/2015/U.S.	Experimental: a randomized clinical trial	312 patients	1,3	To check the effectiveness of the NP care model in patients with chronic pathologies.	The number of readmissions was reduced in the intervention group, and it is very cost‐effective ($311,818 versus $1,019,405).	The NP care model reduced the number of readmissions, apart from turning out to be cost‐effective.	22/25
Maru S/2017/U.S.	Experimental: a randomized clinical trial	624 patients	1,3	To evaluate the effectiveness of a programme led by nurses in the prevention of heart failure in patients at risk.	The number of hospital readmissions was reduced in the intervention group. So far it has not proved to be cost‐effective; a longer evaluation period is needed.	The nursing leadership programme reduced the number of hospital readmissions; however, a longer implantation period is needed to verify if it is cost‐effective.	23/25
Raji M/2016/U.S.	Analytical, retrospective, observational	38,618 patients	1,3	To examine the ratio and the reasons of the change from NP‐exclusive primary care to care only by physicians.	The patients with NPs present fewer readmissions than those under exclusive medical care; besides, this approach is more cost‐effective.	Patient management by the NPs reduces the number of hospital readmissions; thus, this care model turns out to be more cost‐effective than the traditional medical one.	20/22
Agrinier *N*/2013/France	Observational, prospective, analytical	1,222 patients	1,2,3	To evaluate the effectiveness of a heart failure management programme run by nurses so as to check whether the number of readmissions and the costs is lowered for the health system in the Lorraine region.	The patients who participated in the programme presented a lower readmission rate than that of the country (−7.19%). Implementing this programme implied a cost reduction of 1,927,648 euros. Mortality did not vary with respect to the patients who did not participate in the programme.	Apart from being cost‐effective, the nursing leadership programme reduced the number of hospital readmissions.	19/22

## 5 DISCUSSION

The majority of the publications included in the review which analyse in different ways the effectiveness of Advanced Practice Nursing in patients with heart failure come from the U.S. (Kutzleb et al., 2015; Lowery et al., 2012; Maru et al., 2018; Raji et al., 2016; Smith et al., 2015) and from other non‐European countries (Cajanding, 2016; Cheng et al., 2016; De Souza et al., 2014; Stewart et al., 2016), with a clearly visible lack of European publications on this theme (Ågren et al., 2013; Agrinier et al., 2013) since, in European countries, Advanced Practice Nursing is in an uneven and recent level of development: in many of these countries, this strategy is only just beginning to be deployed.

As only four databases are used for the review and, although they are the scientific databases of greatest relevance, it can be deduced that some volume of information may have been lost, although we understand that the application of a review protocol by the two reviewers, and later handling the form of Cochrane Handbook for intervention systematic reviews, has guaranteed the reliability of the review's results.

In spite of the fact that the majority of the selected papers have a high level of evidence, since they are mostly research studies of an experimental or quasi‐experimental character (Ågren et al., 2013; Agrinier et al., 2013; De Souza et al., 2014; Kutzleb et al., 2015; Lowery et al., 2012; Maru et al., 2018; Smith et al., 2015; Stewart et al., 2016), a greater need for experimental studies on this subject is required, as an important volume of excluded articles in the review were narrative bibliographical reviews or opinion articles, with a low level of scientific evidence, contributing only one additional viewpoint on the theme. Nevertheless, it can be concluded that, in general, the quality of the articles included in this review is more than acceptable, considering the impact factor of the journals in which they were published and their level of compliance with the CONSORT and STROBE checklists. In fact, the ranking of the journals with respect to their impact factor is very satisfactory in general, the majority of the journals being in a high quartile, in spite of the fact that two of them are not listed in the JCR (Lowery et al., 2012; Raji et al., 2016).

In this way, to a higher or lower extent, it was possible to answer all the PICO questions, although the evidence level lowered notoriously in the three references exposing research experiences of an observational character, two of them of the retrospective type (Agrinier et al., 2013; Cheng et al., 2016; Raji et al., 2016).

### 5.1 Readmission

Thus, with respect to the reduction or not in the number of hospital readmissions of the patients with heart failure, it can be asserted that all of the papers (Agrinier et al., 2013; Cheng et al., 2016; De Souza et al., 2014; Kutzleb et al., 2015; Lowery et al., 2012; Maru et al., 2018; Raji et al., 2016; Smith et al., 2015; Stewart et al., 2016) agree that, in general, the Advanced Practice Nursing reduce the number of readmissions. In a systematic review of 35 heart failure education studies, the majority demonstrated reduction in readmission after heart failure hospitalization and improvements in patient understanding of heart failure (Boren et al., 2009). The vast majority of the authors point out that the main cause of the improvement in the number of hospital readmissions is determined by the emphasis given to the healthy lifestyles, adequate to the pathological situation of the patient (Agrinier et al., 2013; Cheng et al., 2016; De Souza et al., 2014; Maru et al., 2018; Raji et al., 2016; Stewart et al., 2016), and not so much by the adjustment or prescription of medications (Agrinier et al., 2013; Maru et al., 2018) which, even if beneficial for the patients due to the continuity of care and the higher frequency of them visiting their NPs, are not determinant factors for the improvement posed by this PICO question. Another of the aspects that enables a reduction in this sense is the fast referral to other services or to specialized professionals (Maru et al., 2018), since these health professionals know their own limitations and the patients are always assisted in a service that is adequate to their needs. Even a regular telephone follow‐up produces very beneficial effects when it comes to keeping the patient with heart failure stable (Kutzleb et al., 2015). Therefore, preventing readmission remains one of the key areas of HF management. Consequently, several studies that concentrate on HF readmission have been published, many evaluating multidisciplinary team approaches to HF care (Holland et al., 2005; Takeda et al., 2012). However, evidence relating to more cost‐effective nurse‐led interventions is limited. The concept of hospital readmission is complex. Readmission can be understood as an admission to hospital following a recent discharge. However, the parameters that constitute a readmission vary between studies (Rising et al., 2013). This ambiguity around terminology and different methods of data collection make comparing studies difficult (Au et al., 2012).

### 5.2 Mortality

There is also evidence that Advanced Practice Nursing can improve various and more specific health results than those being evaluated in this bibliographical review, such as blood pressure figures, which diminish in a good number of the articles analysed in this study (Agrinier et al., 2013; Cheng et al., 2016; Stewart et al., 2016).

As regards the reduction or not of the patients’ mortality by means of the Advanced Practice Nursing intervention, there is agreement among the majority of the authors analysed (Cheng et al., 2016; De Souza et al., 2014; Lowery et al., 2012; Stewart et al., 2016), stressing that the mortality of the patients with heart failure decreases considerably or to a lower extent, but that there is always a reduction. However, one author (Agrinier et al., 2013) exposes in his study that the mortality of the patients in the intervention group does not differ from that of the patients in the control group, from where it can be said that the nursing leadership programme analysed in the article does not produce any positive effect in this aspect. The reasons for this reduction in mortality are essentially the same ones given in the first PICO question: emphasis in the change of the lifestyles adequate to the individualized situation of each patient, health education to the patients and their relatives or caregivers, or the correct and more reliable monitoring of the vital constants. Changing the prescription of medications or referring these patients to other specialized services are also other reasons for the reduction of mortality in patients with heart failure.

### 5.3 Cost

It has been established that the prevalence of HF is increasing (Ågren et al., 2012). This increase is due to an ageing population and improved survival after cardiac events (Stewart et al., 2016). This trend is important as HF is associated with significant economic burden. The cost‐benefit ratio of these interventions is another important aspect to be analysed in this review, and it can be said it is positive since a good number of the reviewed studies (Agrinier et al., 2013; Kutzleb et al., 2015; Lowery et al., 2012; Raji et al., 2016; Smith et al., 2015) conclude that Advanced Practice Nursing is cost‐effective when applying interventions to the patient with heart failure; besides, the authors who determine that the interventions performed in their programmes are not cost‐effective (Ågren et al., 2013; Maru et al., 2018)admit that the operation of these same programmes should be assessed for a longer period of time to verify they are not so in the long term, since there is evidence it would be that way.

The cost‐benefit ratio of the health programmes based on Advanced Practice Nursing finds its support on very diverse elements, influencing not only the operation or the type of structure of the health system or of society but also the type of predominant pathologies in the latter and the different academic trainings of the professionals who work in this branch of the nursing profession. Therefore and, although the majority of the authors analysed agree that it is cost‐effective and the rest assert that more time would be needed to verify the economic benefit of their programmes, it is very complicated to transfer a successful working programme or model from one country or region to another.

In one of the studies (Raji et al., 2016) analysed in this review, the number of patients who move from NP care to care of physicians in their medical insurance is investigated, and the results obtained indicate higher hospital readmission values and, as a consequence of the high wages of the medical profession, a reduced cost‐benefit effectiveness; however, it is to be noted that, in this article, the economic benefits in this sense were not studied. Nevertheless, a higher volume of research in this comparative item between these two health professions is needed so as to be able to come to a clear conclusion based on more evidence.

### 5.4 Quality of life

Finally, with respect to quality of life, it could also be concluded that it increases in these type of patients due to the interventions being analysed (Cajanding, 2016; Smith et al., 2015). The reasons are again the same as in the rest of the PICO questions: health and psychosocial education above all and, in the case of one of the articles (Cajanding, 2016), the nursing intervention through a cognitive therapy which reports benefits not only for the patients but also for their relatives and caregivers. The result obtained in one of the articles (Ågren et al., 2013) included in the review is that quality of life does not increase in the intervention group patients when compared to the control group patients; however, it never decreases and sometimes is even lower in the worst‐case scenario. It is because of this that it could be concluded that the answer to this question is positive, although studies could arise more specifically focused on this aspect, maybe addressing longer follow‐ups. However, the data and results related to quality of life should be approached with caution due to the multidimensional nature of the concept.

The results of the specific objectives analysed through the first, second and fourth PICO question are closely related, since they are a definite set of interventions (higher health education emphasis, change of lifestyles, fast referral to specialized services, etc.) that produce a very positive effect on the patient. When the “head” health professional, or the one who really manages the patient, is the nurse, health promotion acquires a really privileged position in the map of actions performed for the benefit of the patient. Studies have been analysed which, although not including the concept of “Advanced Practice Nursing” in their content, do deal with nursing leadership in terms of managing the patient, a concept which has been commented on only a few lines above, and which implies higher control over all the interventions performed on the patient.

### 5.5 Limitations

A search was not performed on grey literature pages or secondary sources. This can lead to increased publication bias.

Consensus was reached by the authors not to use meta‐analysis in this review. A meta‐analysis was not considered feasible owing to the diversity of the primary studies included; a factor that can render meta‐analysis meaningless. The external validity was not measured. The setting of the trials differed; selection of participants; characteristic of participants; hence undermining external validity (Rothwell, 2006).

## 6 CONCLUSIONS

If we understand the effectiveness of Advanced Practice Nursing as the sum of all the concepts analysed in this review, it could be said that this branch of the nursing profession produces very positive effects not only for the patient but also for society as a whole since, in addition to generating positive results in health such as a reduction in the number of hospital readmissions, an improvement in quality of life or the reduction in mortality, it reduces the costs of the health services, enabling economic and social savings within the health system.

It could be considered that all the PICO questions addressed by this review have a positive answer, always with the presence of differences among the individual papers included; however, for this reason, it is to be concluded that a much higher volume of studies would be needed to verify, by means of more evidence, that Advanced Practice Nursing is a branch of the profession capable of facing the new care needs of an ever‐ageing and increasing population, pluripathological and, consequently, polymedicated. It is necessary to further investigate and advance in this field, not only because of the aforementioned but also because of the changes in the health system and its funding, a crucial and motivating aspect which confers and modulates competences to the health professions.

## 7 RELEVANCE FOR THE CLINICAL PRACTICE

This paper represents a descriptive contribution to the usefulness of Advanced Practice Nursing as a developmental strategy in the clinical practice in the management of patients with heart failure. Verifying the impact that this care clinical model can have, largely implemented in the United States and in many other countries but still in development in others, either on mortality, on quality of life or on the reduction of the number of hospital readmissions, and even on the health expenses, can be extremely useful to justify the setting in motion of advanced practice procedures in countries and contexts in which this has not yet been implemented. The demographical and epidemiological changes and the challenge imposed by the economic crises are some of the factors which are facing us with the need to transform the health systems so as to be able to respond to these demands, and the response to heart failure is a concrete example which is requiring us to transform the traditional health care response nurses are providing to these patients into a care model in which nurses show the abilities to adopt complex decisions and the clinical competences needed to offer an enhanced professional practice to this type of complex patient.

In view of the results obtained in this review, advanced practice nurses can contribute to reduce the risk of readmission and death in these patients, improving their quality of life and reducing the associated health costs, reason for which there is a substantial body of international evidence on the positive impact of the Advanced Practice Nursing roles to improve the health results of the patient, the quality of care and the efficiency of the health system, which must lead us to recommend the implementation of this approach in all the health systems aspiring to a universal coverage with the maximum care quality. It has been established that nurses have an important role in comprehensive HF management. Nurse‐led education supports patients in gaining knowledge and through that, empowers patients to become an active participant in their health matters.

## CONFLICT OF INTEREST

The authors declare that they have no conflict of interests.

## AUTHORS' CONTRIBUTIONS

JOP and JAPB contributed to research project elaboration, review of articles, searching of the databases and manuscript writing. JMRR, JGS and NJP contributed to review of articles and manuscript writing. MRM contributed to research project elaboration, manuscript writing and study orientation.

## Data Availability

The data that support the findings of this study are available from the corresponding author upon reasonable request.
